# Impact of Loci Nature on Estimating Recombination and Mutation Rates in *Chlamydia trachomatis*

**DOI:** 10.1534/g3.112.002923

**Published:** 2012-07-01

**Authors:** Rita Ferreira, Vítor Borges, Alexandra Nunes, Paulo Jorge Nogueira, Maria José Borrego, João Paulo Gomes

**Affiliations:** * Department of Infectious Diseases, National Institute of Health, 1649-016 Lisbon, Portugal; † Institute of Preventive Medicine–Faculty of Medicine, University of Lisbon, 1649-028 Lisbon, Portugal, and; ‡ General Directorate of Health, Lisbon, 1049-005 Lisbon, Portugal

**Keywords:** mutation rate, recombination rate, evolutionary inference, ClonalFrame

## Abstract

The knowledge of the frequency and relative weight of mutation and recombination events in evolution is essential for understanding how microorganisms reach fitted phenotypes. Traditionally, these evolutionary parameters have been inferred by using data from multilocus sequence typing (MLST), which is known to have yielded conflicting results. In the near future, these estimations will certainly be performed by computational analyses of full-genome sequences. However, it is not known whether this approach will yield accurate results as bacterial genomes exhibit heterogeneous representation of loci categories, and it is not clear how loci nature impacts such estimations. Therefore, we assessed how mutation and recombination inferences are shaped by loci with different genetic features, using the bacterium *Chlamydia trachomatis* as the study model. We found that loci assigning a high number of alleles and positively selected genes yielded nonconvergent estimates and incongruent phylogenies and thus are more prone to confound algorithms. Unexpectedly, for the model under evaluation, housekeeping genes and noncoding regions shaped estimations in a similar manner, which points to a nonrandom role of the latter in *C. trachomatis* evolution. Although the present results relate to a specific bacterium, we speculate that microbe-specific genomic architectures (such as coding capacity, polymorphism dispersion, and fraction of positively selected loci) may differentially buffer the effect of the confounding factors when estimating recombination and mutation rates and, thus, influence the accuracy of using full-genome sequences for such purpose. This putative bias associated with *in silico* inferences should be taken into account when discussing the results obtained by the analyses of full-genome sequences, in which the “one size fits all” approach may not be applicable.

The ecological success of bacteria relies on their constant ability to diversify their genetic background to reach better-fitted phenotypes through selection. In this regard, point mutations and recombination events are especially relevant as they may be the basis for antigenic polymorphism, virulence dissimilarities, and differential tissue tropism (Ochman *et al.* 2000; Spratt *et al.* 2001; Nunes *et al.* 2010). As for mutation events, in which bacteria range from monomorphic (*e.g. Yersinia pestis*) to highly polymorphic (*e.g.* *Helicobacter pylori*) (Achtman 2008), recombination is not equally important for all microorganisms. Indeed, they range from strictly clonal (lack or extremely low rates of recombination), such as *Mycobacterium* species or *Staphylococcus aureus* (Smith *et al.* 2003; Supply *et al.* 2003; Vos and Didelot 2009), to typical recombinants, such as *Helicobacter pylori* or *Neisseria gonorrheae* (Falush *et al.* 2001; Feil and Spratt 2001). In the middle, there are microorganisms with a moderate recombination background that generate new genomic mosaic structures more fitted to deal with the environment, yielding new successful clones through a never-ending evolutionary process.

The influence of allelic exchange in the evolution of bacterial pathogens has been measured by calculating the relative weight of recombination and mutation rates. Traditionally, these calculations have been performed on multilocus sequence typing (MLST) data resulting from the analysis of housekeeping genes (HK). However, the use of MLST data has yielded strikingly different results within the same species when estimations are performed with dissimilar MLST loci, strain samples, or analytical methodologies (Didelot and Maiden 2010). The rational for using this strategy relies on several arguments. On the one hand, large data sets are available for molecular typing purposes, and HKs are commonly dispersed around the chromosome, which prevents more than one gene from being affected by a single recombination event. Moreover, the use of HKs intends to avoid biased results because the accumulation of mutations may be confounded with the exchange of alleles by recombination when we employ loci that are either “highly polymorphic” or “too conserved”, multicopy or under positive selection (Maiden 2006). Nevertheless, this may not be a straightforward assumption as, except for the fixation of beneficial mutations through positive selection, the occurrence of point mutations exactly in the same genomic position simultaneously for several strains (homoplasy) likely results from recombination within the population (Awadalla 2003). Another question when employing MLST data to infer recombination is the use of a low number of HKs (usually seven), which may not accurately represent the genomic variability. Indeed, a previous study on bacteria found no justifiable reason for applying HKs when inferring intraspecies phylogenetic relationships, and it pointed out that the major concern when choosing candidate loci should rely on their genetic variability (Cooper and Feil 2006). Thus, a wider approach based on using full-genome sequences has been recently applied, as it is expected that biasing effects from “inconvenient” loci are diluted. However, there is a multiplicity of bacterial species in which genomes have a highly heterogeneous representation of loci with different traits, such as polymorphism degree, size of intergenic regions, and selective pressures. Thus, it should be assessed how loci nature shapes the estimation parameters for understanding microbial evolution.

One microorganism that may constitute a good model for evaluating the bias associated with the calculation of recombination and mutation rates through the analysis of different types of loci is the obligate intracellular human pathogen *Chlamydia trachomatis* due to its singular genomic features. Indeed, the core and the pan genomes of the 15 serological variants (serovars) of this pathogen are nearly identical, indicating that horizontal gene transfer is not relevant in *C. trachomatis* evolution. Moreover, the genome similarity among serovars is about 99%, in which major polymorphism is provided by few highly variable loci dispersed throughout the chromosome (Thomson *et al.* 2008), with evidence of positive selection for some of them (V. Borges, A. Nunes, R. Ferreira, M. J. Borrego, and J. P. Gomes, unpublished data; Joseph *et al.* 2011). Also, *C. trachomatis* is under the final stages of the evolutionary process of genome reduction (Zomorodipour and Andersson 1999), containing few nonessential genes and pseudogenes. Therefore, intergenic regions (IGR) likely contain regulatory domains of essential genes, which make IGRs putative targets of selection. In fact, it has been shown that several IGRs exhibit the same phylogenetic signal as neighboring genes (Nunes *et al.* 2008). Finally, although mutation events likely constitute the *C. trachomatis* major evolutionary driving force (Nunes *et al.* 2008), phenomena of genome-dispersed recombination have been recently described, seemingly related to tissue tropism and antigenic variability (Millman *et al.* 2001; Gomes *et al.* 2007; Jeffrey *et al.* 2010). Accordingly, we applied the widely used robust bioinformatic platform ClonalFrame (Didelot and Falush 2007) to several data sets encompassing loci that may differently impact the estimation of recombination and mutation rates, namely, (i) HKs from a recently developed MLST scheme (Dean *et al.* 2009); (ii) positively selected genes (PSG); (iii) five groups of loci strictly ranked by their number of alleles; and (iv) intergenic regions. The results from these data sets were compared with data generated through a wide genomic approach. The present study gets insights on the bias introduced when loci with different genetic features are used to estimate recombination and mutation rates. Our approach differs from previous evaluations (Cooper and Feil 2006; Kennemann *et al.* 2011; Pérez-Losada *et al*. 2006) as we have assessed the individual weight of each group of loci. We believe our results may help to elucidate how the evolutionary parameters are shaped, which will certainly be essential for the comprehension and validation of the data generated through the computational analyses of full-genome sequences.

## Materials and Methods

### Chlamydial culture

By the time this work was performed, only four (A/Har13, B/Jali20, D/UW3, and L2/434) out of the 15 *C. trachomatis* prototype strains (representing the 15 existing serovars) had been fully sequenced (Stephens *et al.* 1998; Carlson *et al.* 2005; Thomson *et al.* 2008; Seth-Smith *et al.* 2009). To obtain sequences for *in silico* analysis, we propagated prototype strains from the remaining serovars (Ba/Apache-2, C/TW3, E/Bour, F/IC-Cal3, G/UW57, H/UW43, I/UW12, J/UW36, K/UW31, L1/440, and L3/404). Our strategy relied on using the 15 prototype strains representing all serovars because tropism differences are well defined at the serovar level, and recent phylogenetic analysis showed that the chosen strains are likely representative of the major genetic variability within the species (Harris *et al.* 2012). Indeed, it is known that differences between same-serovar strains may be as low as 20 single nucleotide polymorphisms (SNP) (Clarke 2011). Cell culture was performed through standard techniques as previously described (Borges *et al.* 2010). Briefly, T_25_ cm^2^ flasks of confluent HeLa 229 cell monolayers were independently inoculated with each strain, and cultures were allowed to grow at 37°, 5% CO_2_ for about 48 hours. After bacterial growth, infected cells were harvested by scraping, sonicating, and centrifuging, and the obtained bacterial pellet was subjected to DNA extraction by using the QIAamp DNA Mini Kit (Qiagen) according to manufacturer’s instructions, and then stored at −80° until use. We then amplified and sequenced the selected genomic regions (see below) for the propagated serovars. PCR primers are listed in supporting information, Table S1. Sequencing was performed as previously described (Gomes *et al.* 2006).

### Loci selection and grouping strategies

**Figure 1:**
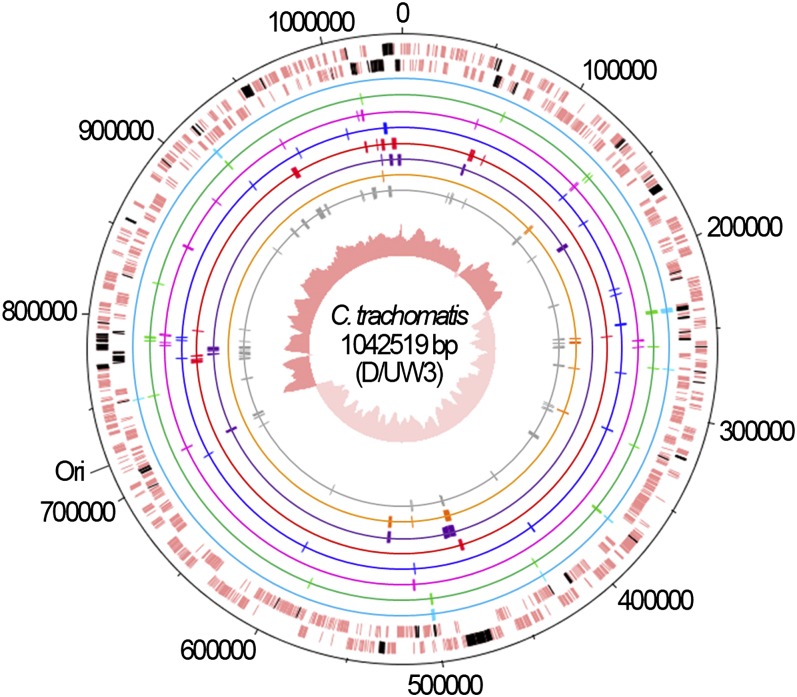
Chromosomal mapping of studied loci. The two outer lanes represent the DNA strands of the *C. trachomatis* chromosome of D/UW3 strain (GenBank accession number NC_000117), where the 80 genes (from the total 136 genomic regions evaluated) are shown in black. Each data set is represented by inner circles: HK-MLST (light blue), alleles 1 to 5 (green), alleles 6 and 7 (pink), alleles 8 and 9 (dark blue), alleles 10 and 11 (red), alleles 12 to 15 (purple), PSG (orange) and IGR (gray). The central circle shows the G/C skew plot. The precise identification of the loci is shown in Table S2.

Considering the high genomic similarity among the *C. trachomatis* serovars (about 99%) (Thomson *et al.* 2008), we used comparative genomics over the four fully sequenced serovars to select informative genomic regions for inferring evolutionary parameters. We were able to select a set of 136 chromosome-scattered and functionally diverse genomic regions (see Table S2), which include 56 IGRs and 80 genes. The selected genomic regions are highly representative of the *C. trachomatis* serovar variability as they comprise about 55% of the total SNPs in just one tenth of the chromosomal length (*P* < 10^−7^) (see Table S3). These regions were then differently grouped according to specific characteristics. First, for each serovar, we created a group encompassing all 136 regions by compiling their sequences while maintaining the relative order of loci in the *C. trachomatis* chromosome. Throughout the text, the strategy using this first data set will be referred to as the wide genomic approach. The second data set, termed HK-MLST, is constituted by the seven HKs that compose a MLST system (Dean *et al.* 2009). Subsequently, we created five additional data sets by dividing the 80 selected genes according to the number of alleles that each gene defines among the 15 *C. trachomatis* serovars: 1 to 5 (17 genes), 6 and 7 (17 genes), 8 and 9 (18 genes), 10 and 11 (15 genes), and 12 to 15 alleles (13 genes) (see Table S2). Finally, we intended to evaluate the impact of using PSGs and IGRs, which are loci categories commonly not recommended when performing this type of analysis, although their potential confounding effects lack experimental support. Thus, we created two data sets composed of 11 PSGs and 56 IGRs, respectively. The use of the IGR data set also relies on recent evidence indicating that noncoding regions may also be affected by selection (Andolfatto 2005; Bush and Lahn 2005) and recombination (Gomes *et al.* 2007), which suggests that there is no apparent reason to completely rule out their use for evolutionary inferences. All studied loci are represented in Figure 1.

### progressiveMauve alignments

Mauve software (http://asap.ahabs.wisc.edu/mauve/) allows the construction of multiple genome alignments for the identification of conserved regions, SNPs, indel events, inversions, and other rearrangements (and their breakpoints location) across the aligned genomes (Darling *et al.* 2004). We aligned the sequences of the 15 prototype strains of each data set through the progressiveMauve algorithm (Darling *et al.* 2010) of the Mauve software v2.3.1. As the sequences length of different data sets were below 1 Mbp, we used a conservative seed weight value (match seed weight = 11) to improve the alignment by reducing noisy matching. The resulting alignments were manually confirmed, and the output files were subsequently used in ClonalFrame software. Although Mauve is particularly useful for aligning full-genome sequences, we used this application as it generates reliable alignments in a compatible format for ClonalFrame.

### ClonalFrame analysis

ClonalFrame (http://www.xavierdidelot.xtreemhost.com/clonalframe.htm) is a widely applied software for inferring the bacterial evolutionary parameters and events underlying DNA sequence variation either from full genomes or from independent regions (such as MLST data sets). The computational cost of the analysis is greatly reduced when the inference is applied to unlinked regions rather than to full genomes, by reconstructing the clonal genealogy and further analyzing each region separately. This is a viable strategy as unlinked regions of the genome are assumed approximately independent given the clonal genealogy of a sample. The ClonalFrame inference is performed in a Bayesian framework, assuming a standard neutral coalescent model (Didelot and Falush 2007).

In this study, the ClonalFrame software v1.2 was used for estimating mutation and recombination rates of dissimilar data sets to evaluate the impact of loci nature on these estimations. Considering the aim of the present study, the ClonalFrame options were selected to (i) estimate the mutation rate (θ), the rate of new polymorphism introduced by recombination (ν), the average tract length of a recombination event (δ), and the recombination rate (R) during each run; (ii) construct a uniformly chosen coalescent tree; (iii) assume a constant population size model; (iv) generate a random seed value for each independent run; and (v) perform the branch swapping attempts in at least half of the time of each iteration. For each data set, two independent ClonalFrame runs were performed. When alignment artifacts hampered the correct function of the software, we manually removed the gap regions while maintaining the genetic variability among *C. trachomatis* serovars, and both new Mauve alignments and ClonalFrame runs were performed. All simulations were carried out using a Linux server.

As different numbers of iterations may yield deviating results, we conducted an analysis of the ClonalFrame reproducibility by performing two independent runs of the wide genomic data set, using a wide range of iterations (30,000, 100,000, 300,000, 500,000, and 1,000,000). For all runs, the first half of the iterations was discarded as burn-ins, and parameters were sampled every 100 iterations during the second half. The optimal number of iterations determined was applied for the subsequent analyses.

We also assessed the convergence of the estimated parameters (θ, R, δ, and ν) from independent runs on the same data set and with the same options by applying the method of Gelman and Rubin (1992) implemented in the Graphical User Interface of the ClonalFrame software. We assumed replicate runs to be convergent only when the calculated test statistic was adequate (*i.e.* below 1.1) for all parameters. Additionally, we performed a fine-tune analysis using the ClonalFrame phylogenetic tree comparison tool, which allows the visualization of the level of confidence (based on a color scale) in each node of the consensus tree of a first run according to the output data of a second run. Each node is given a color code according to the level of confidence; white and black indicate no confidence or total confidence, respectively. On this basis, we attributed a score to each node [ranging from zero (white nodes) to three (black nodes)] (see Figure S1) to achieve a numerical comparison between the runs of different data sets. The sum of the scores of all nodes of each tree was then divided by the respective number of nodes to calculate an average concordance score. Finally, we evaluated the confidence on the estimates of r/m (measure of the weight of recombination on diversification relative to mutation) and ρ/θ (measure of the frequency of occurrence of recombination relative to mutation events) obtained for each data set.

### Nucleotide sequence accession numbers

The sequences of all *C. trachomatis* loci determined in this study were submitted to GenBank under the accession numbers JQ066324–JQ066356 and JQ066367–JQ066722.

## Results and Discussion

The analysis of the evolutionary history of bacteria relies on deciphering genetic differences that arose from several mechanisms, of which point mutations and recombination events are among the most relevant driving forces. The knowledge of the frequency and the relative weight of these two mechanisms is crucial for understanding the biology and the genealogy of microorganisms. This is generally achieved by calculating the ratio ρ/θ, which determines the relative frequency of occurrence of recombination and mutation events, and the ratio r/m, which measures the relative impact of recombination and mutation in genetic diversification. In fact, the estimation of these basic population parameters for microbial pathogens has proved useful, for instance, in explaining the dynamics of drug resistance and pathogenicity and may indicate which epidemiological process should be targeted for disease control (Conway *et al.* 2000; Awadalla 2003). Nevertheless, identifying and determining the exact extent of recombination events is not a simple and straightforward procedure, as there is no ideal methodology for establishing relationships for all bacteria, from strictly clonal to highly recombining microorganisms (Stumpf and McVean 2003). Didelot and Falush (2007) developed a robust computational platform, ClonalFrame, which has yielded valuable results in the inference of both the population structure and the role of the recombination process in several microorganisms, such as *Helicobacter pylori* (Kennemann *et al.* 2011), *Listeria monocytogenes* (Den Bakker *et al.* 2008), and *Salmonella enterica* (Didelot *et al.* 2011). Although most inferences have been generated by using MLST data, it is expected that the analysis of full-genome sequences will be the most applied strategy in the near future. However, loci of different natures are heterogeneously represented in bacterial genomes, and it is not known if they differently impact evolutionary inferences. In the present study, we evaluated how loci nature shapes ρ/θ and r/m estimates, and we used the generated data to speculate about the validity of using full-genome sequences as the approach to estimate such parameters.

### Wide genomic approach

**Figure 2:**
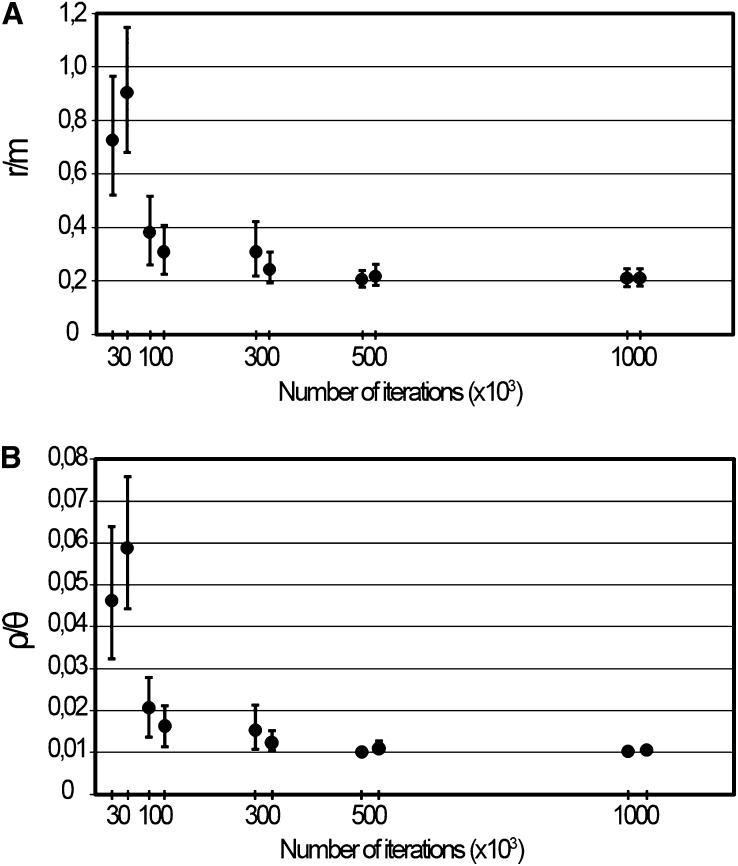
Accuracy assessment of r/m and ρ/θ estimations by varying the number of iterations. The figure illustrates the impact of the number of iterations on the estimations of the ratios r/m (A) and ρ/θ (B) inferred from the wide genomic data set. The graphs present the values and respective 95% confidence intervals of the two independent runs performed with the same number of iterations. The stability (graph plateau), reproducibility (the proximity of the mean estimates from replicate runs), and high levels of confidence (narrower error bars) of both r/m and ρ/θ values were reached only for runs using 500,000 and 1,000,000 iterations.

We compiled loci sequences for all 15 existing serovars, encompassing about 55% of all chromosome SNPs (see Table S3), which is expected to better represent the *C. trachomatis* intraspecies genetic variability. This wide genomic data set was preliminarily used for the assessment of the accuracy of the ClonalFrame analysis by evaluating whether different numbers of iterations (*i.e.* different durations of the simulation period) yield variable results. In fact, the optimization of the number of iterations is a critical step when performing ClonalFrame analysis. The software was run with 30,000, 100,000, 300,000, 500,000, and 1,000,000 iterations for evaluating their impact in both r/m and ρ/θ ratio estimations. We found that the highest dispersion of the estimates of both parameters was obtained for the runs using 30,000 and 100,000 iterations, which noticeably affected the mean values, revealing that for a low number of iterations, small variations may markedly bias the estimation of the evolutionary parameters (Figure 2). By increasing the number of iterations, there was a tendency toward the stability of the results, as similar values were detected when using 500,000 and 1,000,000 iterations. These runs were also the most reproducible and reliable; thus, all subsequent analyses were run by using 1,000,000 iterations to decrease the putative bias strictly associated with simulation duration. We believe that a preliminary step of optimization is critical and mandatory, despite its large computational cost (>50% of the 972 CPU hours dispended in all performed simulations).

**Figure 3:**
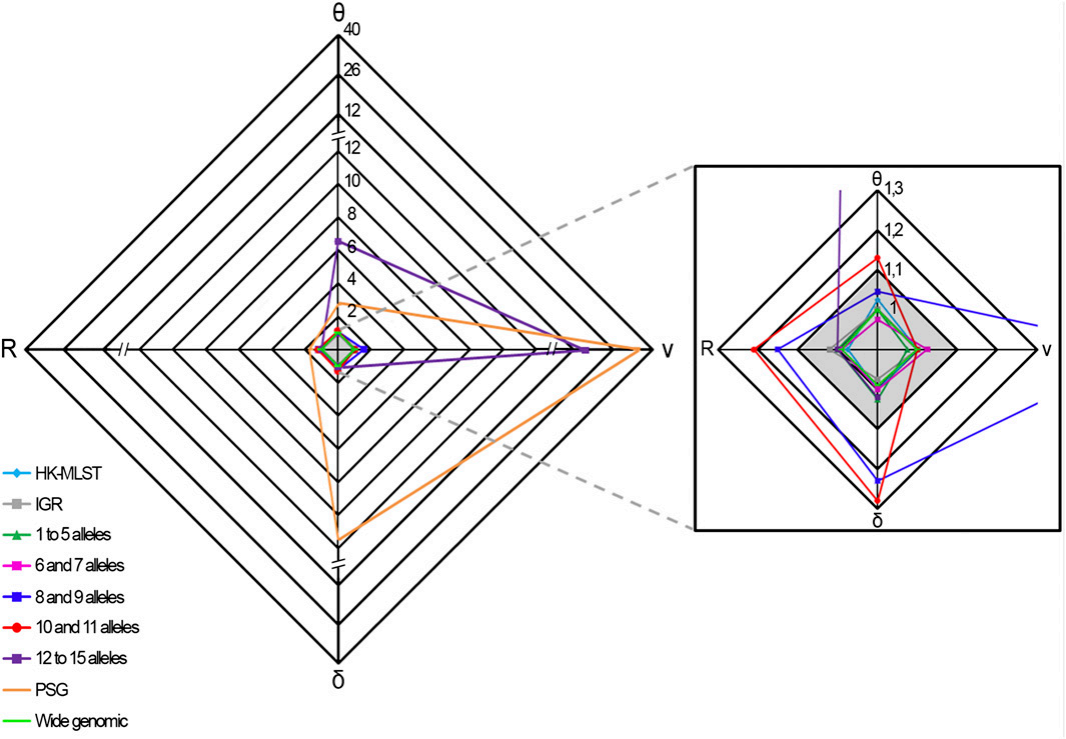
Convergence assessment of the parameters θ, ν, δ, and R. For each data set, the graph shows the convergence values from two independent simulations for the estimated parameters θ, ν, δ, and R. The shaded region of the graph (amplified on the right) indicates the satisfactory range of values (below 1.1) of the test statistic for all parameters according to the Gelman-Rubin test. For the data sets PSG (orange), “8 and 9 alleles” (dark blue), “10 and 11 alleles” (red), and “12 to 15 alleles” (purple), convergence was not observed for at least one parameter.

**Figure 4:**
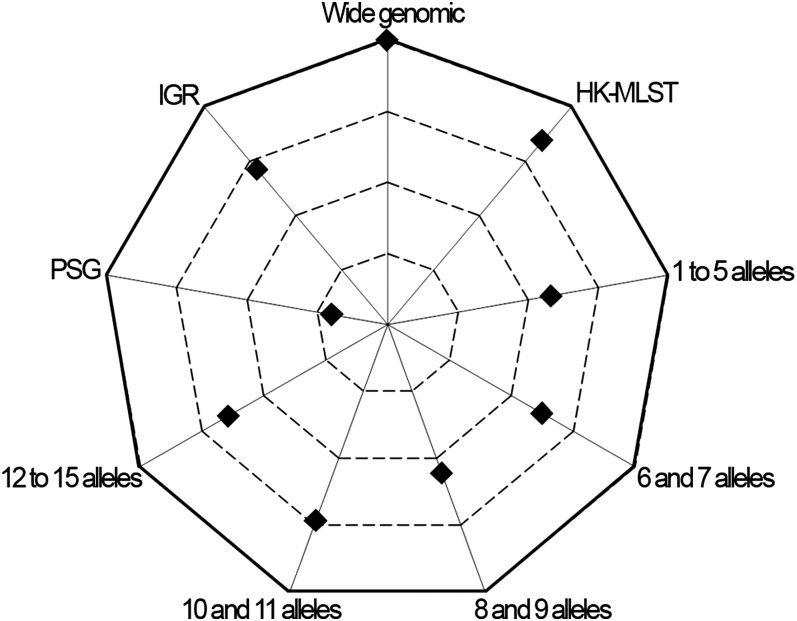
Concordance score between phylogenetic trees. The chart presents the average concordance scores between trees of replicate runs calculated for each data set. More external values correspond to higher concordance between trees, and the outer line represents the maximum average score (score = 3). Values were obtained by using the tree comparison tool of the ClonalFrame, which ranks each node of the first consensus tree according to the level of confidence found between the respective nodes of both trees from replicate runs. The color-based qualitative representation of this tool (see Figure S1) was converted into a quantitative approach as described in *Materials and Methods* to permit the concordance evaluation at the whole-tree level. Only the wide genomic data set reached the maximum average concordance score.

**Figure 5:**
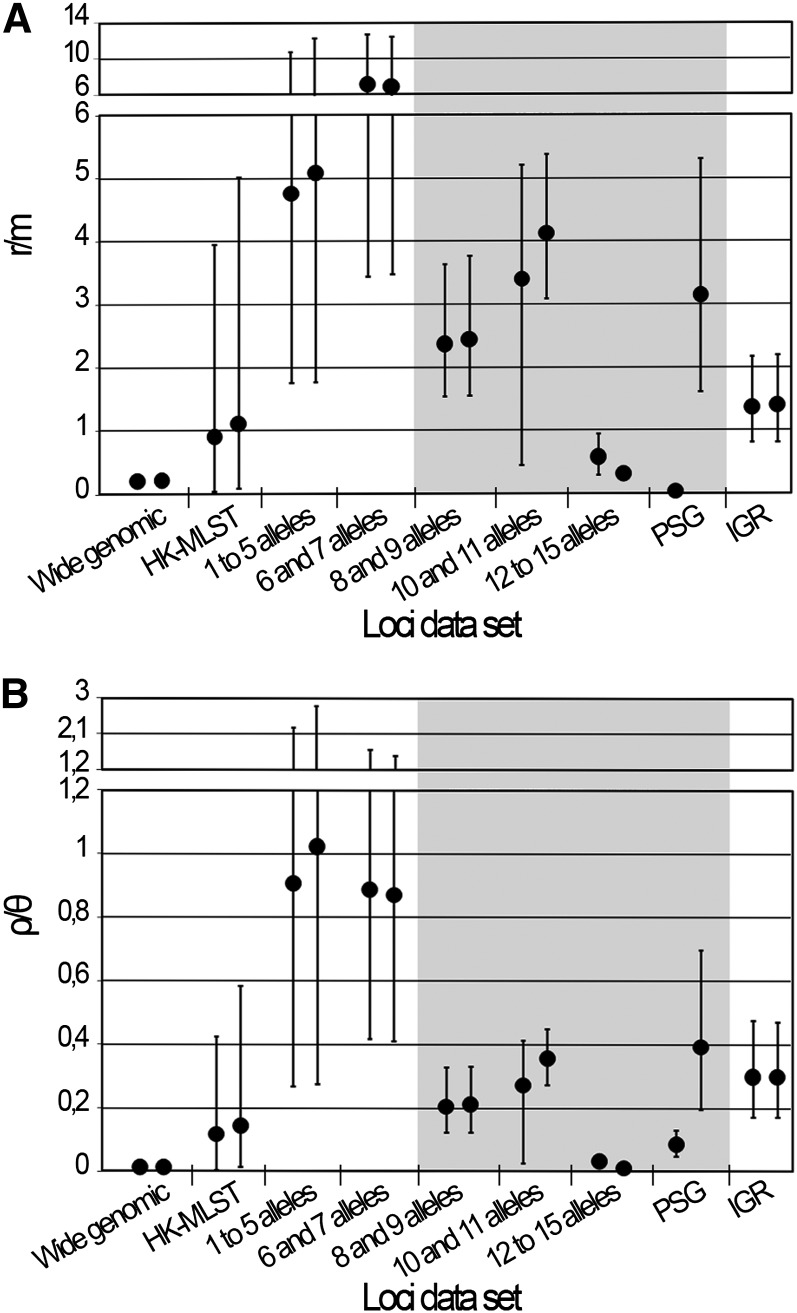
Estimates of r/m and ρ/θ. The graphs show the estimates of r/m (A) and ρ/θ (B) ratios calculated by the ClonalFrame software. For each data set, the results (mean and respective 95% CIs) of the two independent runs performed with 1,000,000 iterations are shown. The data sets that yielded nonconvergent runs assessed by the Gelman-Rubin test (see Figure 3) are shaded in gray.

Another critical stage when estimating r/m and ρ/θ relies on ensuring that independent runs yield convergent estimates for all parameters (θ, R, δ, and ν) and thus sustain similar results. For the wide genomic data set, we observed a convergence scenario for all estimated parameters by using the Gelman-Rubin test implemented in the software (Figure 3, Table S4). As a fine-tune evaluation of convergence, we also used the phylogenetic tree comparison tool, which assesses the degree of concordance between trees from replicate runs (Figure 4, Table S4). It is worth noting that the inferred tree for the wide genomic data set had total confidence in all nodes (average concordance score = 3), which, in addition to the accuracy (Figure 2) and convergence assessment steps, supports that the ratios r/m and ρ/θ were correctly inferred through the analysis of this data set. The mean estimates of r/m and ρ/θ ratios were 0.21 and 0.01, respectively (Figure 5, Table S4), which seem plausible concerning the unique biology of this bacterium. The low ρ/θ value was expected due to the obligate intracellular life style of *C. trachomatis*. Thus, recombination requires a host-cell coinfection by distinct strains [which is expected to occur at a frequency of 1% (Clarke 2011)] followed by the fusion of the inclusion vacuoles where this pathogen replicates. With respect to the low r/m value, the high genomic similarity degree of different serovars (about 99%) implies that, except for well-described situations (Millman *et al.* 2001; Gomes *et al.* 2004, 2006, 2007; Jeffrey *et al.* 2010), a recombinant fragment introduces little diversity in the recipient microorganism. Our estimates using 15 prototype strains are similar to those obtained by Joseph *et al.* (2011) based on four prototype and eight clinical strains (r/m = 0.71 and ρ/θ = 0.07), in which the minor differences may be due to the dissimilar sample sets. Indeed, both results place *C. trachomatis* in the same position (among organisms with low recombination rates) of a r/m “scale” (from 0.02 to 63.6) presented in a previous study that focused on a broad set of bacteria and archaea (Vos and Didelot 2009).

### HK-MLST

Although the MLST data has been widely used for estimating recombination rates of several bacteria, nonconsensual results have been published (Whittam 1995; Feil *et al.* 1999, 2001, 2003; Meats *et al.* 2003), and they may be strikingly conflicting, as illustrated for *Bacillus cereus* in which different studies reported recombination rates differing up to two orders of magnitude (Hanage *et al.* 2006; Pérez-Losada *et al.* 2006). For *C. trachomatis*, a previous study determined a r/m mean estimate of 0.3 based on MLST data (Vos and Didelot 2009), which is in agreement with our estimation using the wide genomic approach, although the authors reported wide 95% CIs (0.0–1.8). Therefore, we decided to test a more recent MLST system (Dean *et al.* 2009) for comparison purposes. We obtained r/m mean values of 1.09 and 0.91, and ρ/θ mean values of 0.12 and 0.14 (Figure 5, Table S4) from convergent and reproducible replicate runs according to both the Gelman-Rubin test (Figure 3, Table S4) and the phylogenetic tree comparison (Figure 4, Table S4), despite the wide CIs that hamper precise estimations (Figure 5, Table S4). Three major issues may underlie the dissimilarity between MLST-based analyses: analytical methodology, strain sampling, and loci selection. As these two analyses using MLST schemes were performed based on ClonalFrame and employed the same set of serovars, we speculate that the loci nature is the major factor influencing estimations. Therefore, MLST data should be applied with prudence when performing this type of evolutionary inference (Achtman 2008), as only a residual proportion of the genome is analyzed [usually 6 to 10 loci of approximately 400 to 600 bp in length (Maiden 2006)], which implies that the whole genetic diversity may not be guaranteed (Didelot and Maiden 2010). This is especially relevant in monomorphic organisms, in which the maximum level of variability is extremely low (Achtman 2008). Nevertheless, the relevance of the application of MLST systems for the characterization of bacterial isolates at the molecular level remains unquestionable.

### Allelic profile

MLST systems usually employ genes that assign a low number of alleles. Therefore, we evaluated the impact of increasing the number of alleles per locus on the estimation of mutation and recombination rates, as the level of polymorphism could shape the results differently. Independent runs were not convergent with the three data sets involving loci that define the highest number of alleles (8 and 9, 10 and 11, and 12 to 15) (*i.e.* Gelman-Rubin statistic above 1.1 for at least one parameter) (Figure 3, Table S4), and thus the parameters are poorly estimated by the software, resulting in inaccurate inferences of r/m and ρ/θ ratios (Figure 5, Table S4). For the two groups of genes assigning a low number of alleles (1 to 5, and 6 and 7), the replicate simulations were convergent and reproducible, but they yielded a high dispersion of both ratios estimates. Moreover, these results contrasted with our estimations using the wide genomic data set and pointed to an implausible scenario of an excessive weight of recombination on genetic diversity of *C. trachomatis* (r/m mean ratios higher than 4 for the two groups) (Figure 5, Table S4). Globally, we found that the level of polymorphism definitely affects the estimations of r/m and ρ/θ at both heterogeneity of results and confidence level. In particular, loci presenting high mutation rates are more prone to confound the estimations, which makes sense considering that an excessive polymorphism is expected to mask the haplotype structures that have evolved over time, making it difficult to analyze the presence or absence of recombination (Awadalla 2003).

### Positively selected genes

The detection of genes under positive selection has been of great importance for clarifying the evolutionary history of bacteria, as they encrypt adaptive signatures that may underlie phenotypic differences, such as those related to pathogenicity (Petersen *et al.* 2007; V. Borges, A. Nunes, R. Ferreira, M. J. Borrego, and J. P. Gomes, unpublished data). However, it has been assumed that PSGs should not be used to infer recombination rates, in spite of the fact that their unsuitability has not been validated experimentally. The rational for their exclusion is that PSGs likely present an unusual number of changes, and the fixation of mutations due to selection could be confounded with their acquisition through a transferred recombining fragment (Maiden 2006). In fact, recombining fragments may bring together beneficial mutations that allow a faster increase in fitness in the presence of major environmental changes instead of solely accumulating point mutations through positive selection (Vos 2009). It is also known that recombination is increased in the proximity of positively selected regions (Vos 2009; Petersen *et al.* 2007), as demonstrated, for instance, for the genus *Streptococcus* (Lefébure and Stanhope 2007). In the present study, we tested a data set composed exclusively of genes putatively under positive selection (Joseph *et al.* 2011; V. Borges, A. Nunes, R. Ferreira, M. J. Borrego, and J. P. Gomes, unpublished data). The evaluation of accuracy revealed lack of convergence for all parameters (values highly above the acceptable cut-off) (Figure 3, Table S4), and the PSG data set was the bottom-ranked group in analysis of the concordance between trees from independent runs (Figure 4, Table S4). Consequently, we found that this data set presented unreliable (wide 95% CIs) and the least reproducible results, which is reflected by the discrepant mean estimate values between runs differing up to two orders of magnitude (Figure 5, Table S4). These results suggest that, for genomes subjected to strong selective pressures, estimations of recombination rates may be biased by the presence of a high fraction of PSGs. Nevertheless, because it is known that PSGs are also targets of recombination (Vos 2009), we believe that, for the majority of the bacterial genomic contexts, the use of wide genome approaches will likely buffer the confounding effects of PSGs on estimations. In fact, in the present study, the inclusion of PSGs in the wide genomic approach did not hamper the accurate inferences of the evolutionary parameters.

### Intergenic regions

The IGRs have been excluded for inferring evolutionary histories of organisms, although they are known to carry promoter regions, ribosome binding sites, as well as transcription factor and regulator binding regions, which play critical roles in regulation of gene transcription. Recent studies demonstrated that noncoding regions are subject to significant selective constraints (Andolfatto 2005; Bush and Lahn 2005). For *C. trachomatis*, we previously detected recombination hotspots involving IGRs (Gomes *et al.* 2007), and we observed phylogenies of IGRs revealing the clustering of strains with the same disease outcomes (Nunes *et al.* 2008), which suggest selection or hitchhiking events (Kaplan *et al.* 1989) involving these regions. This evidence, together with the knowledge that the small genome of *C. trachomatis* likely retains only the indispensable genes (Zomorodipour and Andersson 1999), points to a relevant role of IGRs in *C. trachomatis* evolution. Thus, we estimated rates of recombination and mutation using 56 IGRs because the accumulation of mutations in these regions may not be a random process and because they are heterogeneously represented in different genomes. We obtained >90% of concordance between trees, and a Gelman-Rubin test statistic below 1.1 for all parameters (Figures 3 and 4, Table S4), indicating convergence. The r/m and ρ/θ mean estimates (Figure 5, Table S4) are about 1-log above those obtained for the wide genomic data set, but they are similar to the HK-MLST data set estimates, which suggests that this large set of noncoding regions and these specific HKs shape these evolutionary parameters in a similar fashion for the model under evaluation.

## Conclusion

We used a specific human pathogen with well-defined genomic characteristics as a model to study bias associated with the estimation of evolutionary parameters by computational simulations. Our results show that the estimation of mutation and recombination rates in *C. trachomatis* is influenced by the characteristics of the loci used for such calculations. Although the use of full-genome sequences to infer recombination and mutation rates is suitable for most microorganisms, we anticipate that soon a greater proportion of highly polymorphic or positively selected loci can make it an inaccurate approach. Thus, the correctness of the final output will depend on the dilution effect of these confounding factors by the remaining portions of the genome with dissimilar architectures. As data from population genetics has contributed to a better understanding of the biology and pathogenicity of organisms, the clarification of the putative bias associated with *in silico* inferences is of great interest for deciphering evolutionary traits.

## Supplementary Material

Supporting Information
