# Supplementary material for: Impact of Loci Nature on Estimating Recombination and Mutation Rates in Chlamydia trachomatis
Source: G3 (Bethesda). 2012 Jul 1;2(7):761–8. doi: 10.1534/g3.112.002923 (PMC3385982; doi:10.1534/g3.112.002923)
Supplement: Supporting Information [file supp_2_7_761__index.html]

Supporting Information 

# Impact of Loci Nature on Estimating Recombination and Mutation Rates in *Chlamydia trachomatis*

## Supporting Information for Ferreira *et al.*, 2012

**Files in this Data Supplement:**

- Supporting Information - Figure S1 and Tables S1-S4 (PDF, 2.5 MB)
- Figure S1 - Trees generated by the tree comparison tool of ClonalFrame (PDF, 2.3 MB)
- Table S1 - Oligonucleotide primers used for PCR and sequencing (PDF, 96 KB)
- Table S2 - List of the studied loci (PDF, 86 KB)
- Table S3 - Contingency table for estimating the significance of the polymorphism present in the loci studied (PDF, 59 KB)
- Table S4 - Accuracy results and r/m and ρ/θ estimates for all loci data sets (PDF, 68 KB)
