# Supplementary material for: Impact of Loci Nature on Estimating Recombination and Mutation Rates in Chlamydia trachomatis
Source: G3 (Bethesda). 2012 Jul 1;2(7):761–8. doi: 10.1534/g3.112.002923 (PMC3385982; doi:10.1534/g3.112.002923)
Supplement: Supporting Information [file supp_2.7.761_FigureS1.pdf]

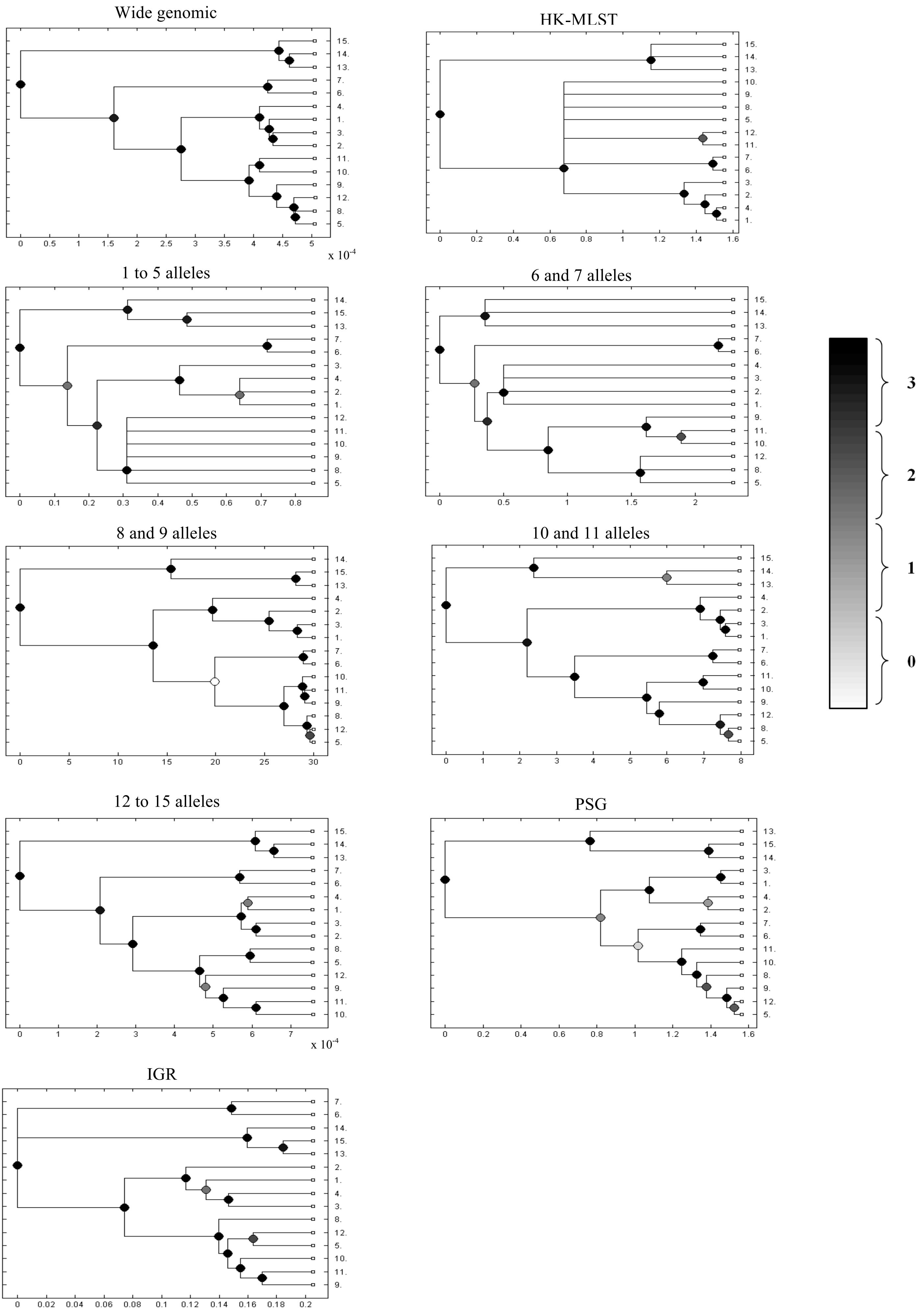
Wide genomic
x 10^-4
HK-MLST
1 to 5 alleles
6 and 7 alleles
8 and 9 alleles
10 and 11 alleles
12 to 15 alleles
x 10^-4
PSG
IGR
3
2
1
0

Figure S1 Trees generated by the tree comparison tool of ClonalFrame. This tool attributes a colored circle to each node of the first consensus tree according to the level of confidence found between both trees of replicate runs (ranging from white, which indicates no confidence, to black, which indicates total confidence). For ranking purposes, a score was given to each node of the resultant tree, ranging from 0 (white nodes) to 3 (black nodes) in order to calculate the average concordance score for each data set. Strains A/Har13, B/Jali20, Ba/Apache2, C/TW3, D/UW3, E/Bour, F/IC-Cal3, G/UW57, H/UW43, I/UW12, J/UW36, K/UW31, L1/440, L2/434 and L3/404 are numbered 1 to 15, respectively.
