# Supplementary material for: Impact of Loci Nature on Estimating Recombination and Mutation Rates in Chlamydia trachomatis
Source: G3 (Bethesda). 2012 Jul 1;2(7):761–8. doi: 10.1534/g3.112.002923 (PMC3385982; doi:10.1534/g3.112.002923)
Supplement: Supporting Information [file supp_2.7.761_TableS1.pdf]

Table S1 Oligonucleotide primers used for PCR and sequencing.

ORF[a]	Primer[a]	Primer sequence (5' to 3')[a]	Primer location[a]	Amplicon size (bp)[a]
CT048 (*yraL*)	CT048-1[b]	GAGCCGGCTCTTTTAAATGGTTT	53104 - 53126	1059
	CT048-2[b]	GTCGACGGAACAGACGAAGAAA	54141 - 54162	
CT050/CT051	CT050/51-1A[c, d]	TGGGCGCTGGTTATTAACTATTTG	377806 - 377829	3704
	CT050/51-2A[b, d]	GACCCCATCCCCTTTGGAGT	381490 - 381509	
	CT050/51-1D[b]	ACAAAGCGCTTTCAGAACATACAT	55452 - 55475	3589
	CT050/51-2D[b]	AGGGCGTCTTTTTCATGATTCTAT	59017 - 59040	
	CT050/51-1[e]	CTAAGAGTTATGTAGCTATC	55542 - 55561	
	CT050/51-1S[e]	AGTTAAGGGAGAGAATCTC	55593 - 55611	
	CT050/51-3[e]	TTGTAGTGTGCAAGATTGTC	55857 - 55876	
	CT050/51-4[e]	TTGTGCCACTACAATACCTT	58726 - 58745	
	CT050/51-5[e]	AACCTTTCCAATATCACCGT	56566 - 56585	
	CT050/51-6[e]	GCACAGATCGCCAATATCAA	58076 - 58095	
	CT050/51-7[e]	AGTCACTCCAGACAATTCTA	57699 - 57718	
	CT050/51-8[e]	TTAGTGAGACAGGCATTGA	57160 - 57178	
CT058/CT059	CT058/9-1[b]	AGTACCGGCCGAATCTCTTTCTCC	67315 - 67338	1634
(*fer*)	CT058/9-2[b]	GTCGGGGGTTCGAATCCCTCTA	68927 - 68948	
	CT058/9-3[e]	TCTCATTACTTCTCTTGCGT	68432 - 68451	
CT147[†]	CT147-1[b]	GGGGAAAGTGAGCTCTTCGGTATC	165430 - 165453	2819
	CT147-2[b]	CGCCGCTACAACAGCTTTAGTGA	168226 - 168248	
	CT147-3[b]	ATTGCGTCCCAAGATATACGACAG	167928 - 167951	2294
	CT147-4[b]	CACGCCAACCCAGAATCCTT	170202 - 170221	
	CT147-5[e]	TAATCATCCACTAGAAGCG	166229 - 166247	
	CT147-6[e]	TGTTCTAGCTGCTCTTGAAT	167649 - 167668	
	CT147-7[e]	TGGATGGTGTTGCAGAATTA	168511 - 168530	
	CT147-8[e]	TACCTCTAGATGTTTTGCGT	169786 - 169805	
CT192	CT192-1[b]	ATATGCGCAAGCACACCTTCC	215717 - 215737	1016
	CT192-2[b]	CTGGGCGTCCATTCACAACA	216713 - 216732	
	CT192-3[e]	CGTATCGATTCCTTCTTCTA	215998 - 216017	
	CT192-4[e]	CTCCTCTTATTGAAGAAGCT	2156196 - 216215	
CT195	CT195-1[b]	CCTCCGCCTAATCCTCGACTACAT	219655 - 216678	1320
	CT195-2[b]	CCAGCGGTTGATATTTCTTGATTA	220951 - 220974	
CT214	CT214-1[b]	AGGGCTTCTATTCCTCAAACAGTA	241463 - 241486	1797
	CT214-2[b]	TTCCCCGTTCTAAAGATCAGTTAT	243236 - 243259	
	CT214-3 [e]	AACAGCCTGGATCTATATCA	242027 - 242046	
CT223	CT223-1[b]	GCAACGCATATCGCTCCTCA	251102 - 251121	1308
	CT223-2[b]	GTGCGCCCCTTCTCGTAAAG	252390 - 252409	
CT228/CT229	CT228/9-1[b]	CGGTCCCGGATTATCAAAACAAGT	254667 - 254690	1816
	CT228/9-2[b]	ATGCGGCCATCCCAGAAGC	256464 - 256482	
	CT228/9-3[e]	AGATTACGCAAACGTTGCTC	255033 - 255052	

	CT228/9-4[e]	GTTGTGATTGCAGCAGTAG	255972 - 255990	
CT232/CT233	CT232/3-1[b]	GATTAGGCGGAGGGGTTCTCTT	259223 - 259244	1262
(*incB*/*incC*)	CT232/3-2[b]	CTCTCCGCGACGCAAACTAAG	260464 - 260484	
CT249	CT249-1[b]	ACCACCCTTTAGCCATCCATTCC	279170 - 279192	704
	CT249-2[b]	AATTGCGCCGCCTCCTTGTA	279854 - 279873	
CT288	CT288-1[b]	TTTTACGCACAATGAACCCAGAAA	321582 - 321605	2063
	CT288-2[b]	CGGGCTCCTCGGGAACAG	323627 - 323644	
	CT288-3[e]	TTACCTGACCTCAGACACC	322164 - 322182	
	CT288-4[e]	GTCAGCTCGTCGTTTATTG	323121 - 323139	
CT293 (*accD*)	CT293-1[b]	TGCGCCAGAAGCTCCAGAAGTAGC	326322 - 326345	1350
	CT293-2[b]	AGGATCTGGCTGGGGATGGTTAGC	327648 - 327671	
CT365	CT365-1[b]	AAATTCGCAAACTTGCTCTTTTTC	416102 - 416125	2213
	CT365-2[b]	GATCGGGATTCCCCTGGATA	418295 - 418314	
	CT365-3[e]	CTAACTTCCAAGTTTCCTCT	416572 - 416591	
	CT365-4[e]	CTCATTGCAGGTATTGTTGT	417720 - 417739	
CT442 (*crpA*)	CT442-1[b]	CTCCTCCCTTTCCATACATCATCT	511256 - 511279	783
	CT442-2[b]	AAGCGATTCTTTCTTCCGATACAT	512015 - 512038	
CT456 (*tarp*)	CT456-1[b]	ACAAACGTTACCCGGTATGCTGTT	530723 - 530746	3362
	CT456-2[b]	TTGCGCCTTGTCGATTGTGAT	534064 - 534084	
	CT456-3[e]	TACCTCATCAAGCGATCATA	531252 - 531271	
	CT456-4[e]	CCACCAGTTGTTATTATGTC	533470 - 533489	
	CT456-5[e]	AGACATGTCTCTTCCTTCAT	531867 - 531886	
	CT456-6[e]	TACATCAGAGATTACGTCTC	532889 - 532908	
	CT456-7[e]	GAGTTTCATTGGAGAAGGAA	532413 - 532432	
	CT456-8[e]	CGTTACCCGGTATGCTGTT	530728 - 530746	
	CT456-9[e]	TACAAACACTACTGCCTTCA	533363 - 533382	
	CT456-10[e]	TTGTTACTACCTACGTCATC	531328 - 531347	
	CT456-11[e]	CTAATTAAATCGGCTGTTG	530869 - 530887	
CT529	CT529-1[b]	ACGCGGCTCCTTAAAGCAAACAA	596464 - 596486	1659
	CT529-2[b]	CGCGCATATCCGGGGAGTCT	598103 - 598122	
	CT529-3[e]	TCTCGCAAGCATTTTCCTCT	596984 - 597003	
CT618	CT618-1[b]	TCCCGATATGCCTCCTTTTGAGTC	698080 - 698103	1360
	CT618-2[b]	ATGCGCACGCAAGCCAATC	699421 - 699439	
CT622	CT622-1[b]	GGCTCCCCCTCAATTCACAAACTT	707046 - 707069	2319
	CT622-2[b]	GGTCGCGGAAACCAAATGAAATA	709342 - 709364	
	CT622-3[e]	TGATTGCTTGTATTTCGGCT	707784 - 707803	
	CT622-4[e]	TTCAGCATCGTCCTCTGTAA	708885 - 708904	
	CT622-5[e]	AGAAGAGATTATGCAGAAGC	707298 - 707317	
CT623	CT623-1[b]	TTTGCCCATTAATAATTGGATTCA	708957 - 708980	1516
	CT623-2[b]	CATGGGTCGGTTGTATGAGATGT	710450 - 710472	
CT653 (*yhbG*)	CT653-1[b]	AGCCGCGATAGCTAACGAAGTG	750194 - 750215	922
	CT653-2[b]	GAAGGCGGAATGAAAGTCCTCTC	751093 - 751115	

CT674 (*yscC*)	CT674-1[b]	TTTCAAGCGGAATCGCAAGGAAT	770234 - 770256	3229
	CT674-2[b]	CCGGGATCGAACCGACGAC	773444 - 773462	
	CT674-3[e]	AGAGCCATCAGATTTTCTCT	770846 - 770865	
	CT674-4[e]	AGAGGAAGAGAACTGAGTAA	772996 - 773015	
	CT674-5[e]	ATGACTTGAAAGTCGTTGAA	771470 - 771489	
	CT674-6[e]	TCCGTACATCATATCACTGA	772505 - 775524	
	CT674-7[e]	GATATCGGAGTCAATCTTGTT	772771 - 772791	
CT675 (*karG*)/ CT676	CT675/6-1[b]	CCCGGCTTTGGGCATTCC	773458 - 773475	1933
	CT675/6-2[b]	TCATTCCGGTAACAGGGGTTCG	775369 - 775390	
	CT675/6-3[e]	CGTGATCAGATTAATCAGCT	774669 - 774688	
CT677/CT678/ CT679 (*frr, pyrH, tsf*)	CT677/9-1[b]	ATGGGGCCAGGACGGGTCTA	775420 - 775439	2393
	CT677/9-2[b]	AAATTTTATCTCCGGTGCGTCCTG	777789 - 777812	
	CT677/9-4[e]	GAAGATATCACTGTACCAAC	776088 - 776107	
	CT677/9-5[e]	TGGCTAAAGACATTGCTATG	777107 - 777126	
CT682 (*pbpB*)[f]	CT682-1[b]	GCATTGTGATCGCGCAGGAGTA	780841 - 780862	3149
	CT682-2[b]	TTTCCGCCTCTTCCATAGTCGTTC	783966 - 783989	
	CT682-3[e]	TTGATAGGCAAGCGATCTAT	781484 - 781503	
	CT682-4[e]	ATATTCTCCAGGAAGTCCTA	783318 - 783337	
	CT682-5[e]	TGGAAATGTTTGAGTGTGAA	782079 - 782098	
CT683 (*TPR-motif protein*)	CT683-1[b]	TCGCTGCGGTAGGATATGAAGATG	783756 - 783779	1702
	CT683-2[b]	TCGCCGCGTAAATGAACCAAT	785437 - 785457	
	CT683-3[e]	GACTGTCAAACAGCCCTTAA	785108 - 785127	
CT684[f]	CT684-A1[b]	TGGGCATTACAATCTTGGGTTATG	784615 - 784638	1471
	CT684-A2[b]	AACAGCGGCATGCAGTTGATG	786065 - 786085	
	CT684-B1[b]	ATTTCGGGAGGCAATCCACAAT	785785 - 785806	1398
	CT684-B2[b]	TCCCGGGAATCCATATACCTCTTC	787159 - 787182	
	CT684-A3[e]	TCATCAATAGGGATGCCTAA	785651 - 785670	
	CT684-A4[e]	TCTGGATCTGCGTCTTCTAA	785609 - 785628	
CT685/CT686	CT685/6-1[b]	GCTCGGGAAGCAACCAAGTTATTA	786722 - 786745	2386
	CT685/6-2[b]	AAGCGAGTTCCCATGATACGAGAT	789084 - 789107	
	CT685/6-3[e]	TCTTGATGTCGATGCTTTGA	787308 - 787327	
	CT685/6-4[e]	ATGCAGAAGCTCGATTACTT	788502 - 788521	
CT694	CT694-1[b]	TACAGGGGGAGGCGCTTCCTTA	796071 - 796092	1541
	CT694-2[b]	CGCGCTCTTCTAGCTCTCCCTCTT	796528 - 797551	
	CT694-3[e]	GATAACTCTTAACCCCATTG	796267 - 796286	
CT760 (*ftsW*)	CT760-1[b]	CTTGGGCCATTGCATTGAGTAAT	892695 - 892717	1417
	CT760-2[b]	CCCCAGAGAACATCCGATTGAC	894090 - 894111	
	CT760-3[e]	AGGATGTAGGTAAACTTGCA	893445 - 893464	
CT783	CT783-1[b]	AGCGGGGATTCAGCATTCCT	919328 - 919347	1427
	CT783-2[b]	TGCCCCTCGCCTCTTCATC	920676 - 920694	
	CT783-3[e]	TGTCAATACCTTCCCTAGTT	919913 - 919932	
CT813	CT813-1[b]	CTGCGTGTCGCTCTGGAAAATAAT	954988 - 955011	1460

	CT813-2[b]	AGGCCGAGCCCTACTCAAAAACT	956365 - 956387	
CT818 (*tyrP_2*)	CT818-1[b]	CCTGGCGGGAAAGGGACTCT	961600 - 961619	1531
	CT818-2[b]	GCGCATAATCGCGATCATACAATC	963107 - 963130	
	CT818-3[e]	GAATAGCACGTTTAACCTCA	962512 - 962531	
CT852 (*yhgN*)	CT852-1[b]	CTGCCGCACCAGCAAGGAT	1001213 - 1001231	768
	CT852-2[b]	TAGGCGCTCAACTTCTGGTATCTG	1001957 - 1001980	
CT859 (*ispH*)/	CT856/60-1[b]	GAGGGGGCTTTGCGGATTTAT	1011689 - 1011709	2779
CT860	CT856/60-2[b]	CCGGAATGCTTGGCTTGACA	1014448 - 1014467	
	CT856/60-3[e]	ACGACATTGAGTATGGATGA	1012253 - 1012272	
	CT856/60-4[e]	ACCCCGATATCTCATAAATC	1013829 - 1013848	
	CT856/60-5[e]	GTATTTCAGTTGCCTAAGGA	1012658 - 1012677	
CT861/CT862	CT861/2-1[c]	GAGGGCAGAGGCTTCTTCACAAG	1014122 - 1014144	2400
(*lcrH_2*)	CT861/2-2[b]	CTAGGCGTCCCAATTGGAGACTC	1016499 - 1016521	
	CT861/2-3[e]	AATAGCTCTCCAACCATCAA	1014640 - 1014659	
	CT861/2-4[e]	GACTATGAGGAAAGTTCTAC	1016071 - 1016090	
	CT861/2-5[e]	TCTCCTGTTGCTATTGTTTG	1014159 - 1014178	
	CT861/2-6[e]	TTGACTTTCTCCTGATGCTT	1015102 - 1015121	
CT867/CT868	CT867/8-1[c]	TCCCGACTGCTGGGGCTTAGA	1022902 - 1022922	2889
	CT867/8-2[c]	CATCGCGTCATGCCATGTCCTAT	1025768 - 1025790	
	CT867/8-3[e]	TCCTTCAGCTACTCTGATT	1022935 - 1022953	
	CT867/8-4[e]	TGGTAAGCGGATTACAGAT	1023641 - 1023659	
	CT867/8-5[e]	TTCATGGCGTTCTACAGAAT	1024302 - 1024321	
	CT867/8-6[e]	AATGTCAGAATCCCAAGCA	1024923 - 1024941	
	CT867/8-7[e]	AGAGGGAAGTCTTAATTTCC	1025542 - 1025561	

[a] Open reading frame (ORF) numbers, primer location (genome coordinates), primer sequences and amplicon size are based on the D/UW3 strain genome annotation (GenBank accession number NC_000117).

[b] Amplification primers also used for automated sequencing.

[c] Primers exclusively used for PCR amplification.

[d] Primer sequences, location and amplicon size refers to the L2/434 strain genome annotation (GenBank accession number NC_010287) as these primers were designed for amplification and automated sequencing in LGV and ocular strains, and they have no homology in the D/UW3 genome sequence.

[e] Primers exclusively used for automated sequencing.

[f] Due to the large gene size (for CT147 and CT682) or for PCR optimization (for CT684), two PCR primer pairs were designed to generate two overlapping amplicons for the entire gene. The primer pair for the amplification of the first region of the CT682 gene was previously described in Gomes et al. 2007 (data not shown).
