# Supplementary material for: Impact of Loci Nature on Estimating Recombination and Mutation Rates in Chlamydia trachomatis
Source: G3 (Bethesda). 2012 Jul 1;2(7):761–8. doi: 10.1534/g3.112.002923 (PMC3385982; doi:10.1534/g3.112.002923)
Supplement: Supporting Information [file supp_2.7.761_TableS2.pdf]

Table S2 List of the studied loci.

Loci [a]	Alleles Number [b]	$d_N/d_S > 1$ (Z-test; p <0,05) [c]
IGR (CT044/CT045)	-	
IGR (CT047/CT048)	-	
CT048 (*yraL*)	6	
IGR (CT048/CT049)	-	
CT049	13	
CT050	11	
CT051	11	
CT058	11	
IGR (CT058/CT059)	-	
CT059 (*fer*)	5	
CT115	6	
IGR (CT115/CT116)	-	
CT116	8	+
CT117	7	
IGR (CT117/CT118)	-	
CT118	5	+
IGR (CT118/CT119)	-	
CT121 (*araD*) [d]	3	
CT144	9	
IGR (CT144/CT145)	-	
CT147 (*EEA1*)	12	
IGR (CT191/CT192)	-	
CT192	8	
IGR (CT192/CT193)	-	
CT195	8	
CT209 (*leuS*) [d,e]	5	
CT214	8	
IGR (CT222/CT223)	-	
CT223	11	+
IGR (CT223/CT224)	-	
CT228	6	+
IGR (CT228/CT229)	-	
CT229	7	+
IGR (CT229/CT230)	-	
IGR (CT231/CT232)	-	
CT232 (*IncB*)	5	
IGR (CT232/CT233)	-	
CT233 (*IncC*)	7	
IGR (CT233/CT234)	-	
CT245 (*pdhA*) [d,e]	2	

IGR (CT248/CT249)	-	
CT249	9	+
IGR (CT287/CT288)	-	
CT288	9	+
IGR (CT288/CT289)	-	
IGR (CT292/CT293)	-	
CT293 (*accD*)	4	
IGR (CT293/CT294)	-	
IGR (CT315/CT316)	-	
CT332 (*pykF*) [d,e]	5	
CT365	8	
IGR (CT365/CT366)	-	
CT376 (*mdhC*) [d,e]	4	
CT412 (*pmpA*)	10	
CT413 (*pmpB*)	13	+
IGR (CT413/CT414)	-	
CT414 (*pmpC*)	13	
CT432 (*glyA*) [d,e]	3	
IGR (CT441/CT442)	-	
CT442 (*crpA*)	8	+
IGR (CT442/CT443)	-	
CT443 (*omcB*)	7	
CT456 (*tarp*)	13	+
CT505 (*gapA*) [d]	3	
CT529	9	
IGR (CT529/CT530)	-	
CT618	8	
IGR (CT618/CT619)	-	
CT622	12	
IGR (CT622/CT623)	-	
CT623	6	
IGR (CT624/CT625)	-	
IGR (CT652.1/CT653)	-	
CT653 (*yhbG*) [e]	3	
IGR (CT653/CT654)	-	
CT674 (*yscC*)	11	
IGR (CT674/CT675)	-	
CT675 (*karG*)	10	
CT676	8	
IGR (CT676/CT677)	-	
CT677 (*frr*)	10	
CT678 (*pyrH*)	10	
IGR (CT678/CT679)	-	
CT679 *(tsf*)	12	

CT680 (*rs2* or *rpsB*)	12	
IGR (CT680/CT681)	-	
CT681 (*ompA*)	15	
IGR (CT681/CT682)	-	
CT682 (*pbpB*)	14	
IGR (CT682/CT683)	-	
CT683 (*TPR-motif protein*)	7	
IGR (CT683/CT684)	-	
CT684	6	
CT685	5	
CT686	9	
CT687 (*yfhO_1*)	5	
IGR (CT687/CT688)	-	
CT688 (*parB*)	8	
CT689 (*dppF*)	6	
CT690 (*dppD*) [d]	6	
CT694	10	+
IGR (CT698/CT699)	-	
CT713 (*porB*) [d]	4	
16SrRNA	6	
IGR (CT759/CT760)	-	
CT760 (*ftsW*)	6	
CT781 (*lysS*) [d,e]	4	
IGR (CT782/CT783)	-	
CT783	8	
IGR (CT783/CT784)	-	
IGR (CT796/CT797)	-	
CT812 (*pmpD*)	10	
IGR (CT812/CT813)	-	
CT813	7	+
IGR (CT813/CT814)	-	
IGR (CT817/CT818)	-	
CT818 (*tyrP_2*)	9	
IGR (CT818/CT819)	-	
IGR (CT851/CT852)	-	
CT852 (*yhgN*)	9	
IGR (CT852/CT853)	-	
CT859 (*ispH*)	6	
IGR (CT859/CT860)	-	
CT860	10	
IGR (CT860/CT861)	-	
CT861	7	
IGR (CT861/CT862)	-	
CT862 (*lcrH_2*)	3	

CT867	10	+
CT868	13	+
CT869 (*pmpE*)	11	
CT870 (*pmpF*)	9	
IGR (CT870/CT871)	-	
CT871 (*pmpG*)	12	
CT872 (*pmpH*)	10	
CT874 (*pmpI*)	12	

[a] Open reading frame (ORF) numbers are based on the D/UW3 strain genome annotation (GenBank accession number NC_000117).

[b] Number of alleles that each gene assigns were determined based on MEGA5 evolutionary analysis (distance matrices and phylogenetic trees) of each gene (for more details see the Materials and Methods section).

[c] Genes putatively under positive selection that compose the PSG data set (Joseph et al. 2011; Borges V, Nunes A, Ferreira R, Borrego MJ, Gomes JP, unpublished data)..

[d] For these genes, the partial sequences available at the GenBank were used.

[e] Genes that constitute a previously defined MLST scheme for *Chlamydia trachomatis* molecular characterization (Dean et al. 2009). The partial sequences used for typing purposes compose the HK-MLST data set.
