# Supplementary material for: Impact of Loci Nature on Estimating Recombination and Mutation Rates in Chlamydia trachomatis
Source: G3 (Bethesda). 2012 Jul 1;2(7):761–8. doi: 10.1534/g3.112.002923 (PMC3385982; doi:10.1534/g3.112.002923)
Supplement: Supporting Information [file supp_2.7.761_TableS3.pdf]

Table S3 Contingency table for estimating the significance of the polymorphism present in the loci studied.

	Inside Region (bp) [a]	Outside Region (bp)	
SNPs	1802	1552	3354 [b]
Without SNPs	112530	926635	1039165
	114332	928187	1042519 [c]

[a] Chromosomal region evaluated in the present study (composed by the 136 genomic regions: 80 genes and 56 intergenic regions). [b] Total number of SNPs identified between the *Chlamydia trachomatis* serovars A/Har13 (NC_007429) and D/UW3 (NC_000117) (Carlson et al. 2005).[c] Total length of the chromosome of *C. trachomatis* serovar D/UW3 (NC_000117). The polymorphism significance ($p < 10^{-7}$) of the analyzed regions was calculated using the Fisher's exact test.
