# Supplementary material for: Impact of Loci Nature on Estimating Recombination and Mutation Rates in Chlamydia trachomatis
Source: G3 (Bethesda). 2012 Jul 1;2(7):761–8. doi: 10.1534/g3.112.002923 (PMC3385982; doi:10.1534/g3.112.002923)
Supplement: Supporting Information [file supp_2.7.761_TableS4.pdf]

Table S4 Accuracy results and r/m and ρ/θ estimates for all loci data sets.

Loci Data Sets	Accuracy Assessments			ClonalFrame Results			
	Trees concordance score	Gelman-Rubin test		r/m (mean; [95% CI])		ρ/θ (mean; [95% CI])	
		Test statistics	Convergence	1st run	2nd run	1st run	2nd run
Wide genomic	3	1.00 (θ, ν, δ); 0.99 (R)	+	0.21; [0.18-0.25]	0.21; [0.18-0.24]	0.01; [0.01-0.01]	0.01; [0.01-0.01]
HK-MLST	2.875	1.00 (θ, ν, δ, R)	+	0.91; [0.05-3.95]	1.09; [0.07-5.02]	0.12; [0.004-0.42]	0.14; [0.01-0.58]
1 to 5 alleles	2.667	1.00 (θ, δ, R); 0.99 (ν)	+	4.77; [1.77-10.72]	5.09; [1.76-12.36]	0.91; [0.27-2.27]	1.02; [0.27-2.87]
6 and 7 alleles	2.7	1.00 (δ, R); 0.99 (θ, ν)	+	7.04; [3.45-12.67]	6.98; [3.47-12.57]	0.89; [0.42-1.71]	0.87; [0.41-1.62]
8 and 9 alleles	2.643	1.04 (θ); 1.15 (R); 1.58 (ν); 1.23 (δ)	-	2.38; [1.54-3.65]	2.44; [1.55-3.77]	0.21; [0.12-0.33]	0.21; [0.12-0.33]
10 and 11 alleles	2.786	1.13 (θ); 1.21 (R); 1(ν); 1.28 (δ)	-	3.41; [0.47-5.21]	4.12; [3.09-5.38]	0.27; [0.03-0.41]	0.35; [0.27-0.45]
12 to 15 alleles	2.714	6.51 (θ); 1(R); 16.99 (ν); 1.02 (δ)	-	0.58; [0.3-0.96]	0.29; [0.21-0.38]	0.03; [0.01-0.05]	0.01; [0.01-0.01]
PSG	2.357	2.70 (θ); 1.73 (R); 37.74 (ν); 11.45 (δ)	-	0.05; [0.02-0.1]	3.15; [1.6-5.31]	0.08; [0.05-0.13]	0.39; [0.2-0.7]
IGR	2.769	1.00 (θ, ν, δ, R)	+	1.38; [0.82-2.17]	1.38; [0.8-2.19]	0.3; [0.17-0.48]	0.29; [0.17-0.47]

CI – Confidence interval; **θ** – mutation rate; ***ν*** – rate of new polymorphism introduced by recombination; **δ** – average tract length of a recombination event; **R** – recombination rate; **r/m** – measure of the weight of recombination on the diversification relative to mutation; **ρ/θ** – measure of the frequency of occurrence of recombination relative to mutation events.
